# Junior athletes’ nutritional demands: a narrative review of consumption and prevalence of eating disorders

**DOI:** 10.3389/fnut.2024.1390204

**Published:** 2024-09-24

**Authors:** Adam Amawi, Batool Khataybeh, Raghad Al Aqaili, Nour Ababneh, Lana Alnimer, Ali Qoqazeh, Farah Oukal, Haitham Jahrami, Khitam Mousa Ay, Hassan Al Saoud, Hadeel Ghazzawi

**Affiliations:** ^1^Department of Exercise Science and Kinesiology, School of Sport Sciences, The University of Jordan, Amman, Jordan; ^2^Department of Nutrition and Food Technology, Faculty of Agriculture, Jordan University of Science and Technology, Irbid, Jordan; ^3^Department of Nutrition and Food Technology, School of Agriculture, The University of Jordan, Amman, Jordan; ^4^Department of Nutrition and Food Processing, School of Agriculture, Al-Balqa Applied University, Al-Salt, Jordan; ^5^Government Hospitals, Manama, Bahrain; ^6^Department of Psychiatry, College of Medicine and Medical Sciences, Arabian Gulf University, Manama, Bahrain

**Keywords:** dietary requirements, junior athletes, performance, macronutrients, supplements, food choices, eating disordered junior, “Cadet”

## Abstract

Information regarding the dietary requirements and consumption of young athletes is limited. Hence, the aim of this narrative review is to provide a comprehensive combination of research and review papers on the nutritional status of young athletes aged 5–18 years old, as well as quantitative, qualitative, wholesome foods, food choices, and eating disordered data concerning the dietary requirements for growing young athletes. This study involved systematic searches of electronic databases, including Google Scholar, PubMed, Science Direct, Scopus, and Web of Science. The specific criteria for identifying research papers published in English from July 1980 until May 2024 were included. Only 48 studies out of 1,262 were included in this narrative review. The findings of this study suggest that, compared with adults, junior athletes need a unique approach to meet their dietary needs. Growth, development, and general athletic performance depend on macronutrients, as they are vital nutrients for young active athletes. However, research on enhancing junior athletes’ performance is still in progress, and studies on hydration status, and eating disorders are limited.

## 1 Introduction

The media in general and the sports media in particular have purposeful roles in disseminating new training methodologies, which has raised the competition level for athletes from an early stage of their lives (1). The nutritional treatment of young athletes is an interesting scientific field that combines exercise physiology and nutritional sciences. Despite the high level of competition between young and adult athletes, we cannot follow a single diet for both groups, as there are many differences in substrate storage and substrate metabolism, apart from physiological changes in the growth stages (2). Poor counseling among young athletes causes them to make food choices that have a negative impact on their health and athletic performance; these choices include skipping meals, eating fast food, and engaging in high-risk behaviors such as dehydration techniques, self-induced vomiting, restrictive eating, over exercising, using diuretics, using laxatives, or eating diet medications (3, 4).

Recent studies highlight the importance of consuming a well-balanced diet containing both macronutrients and micronutrients as proper athlete nutrition for children and adolescents for stimulating training, increasing physical activity, reducing fatigue during exercise, and preventing injuries. The muscle mass of young athletes is also maintained by providing proper daily energy, macronutrients, and micronutrients according to several physiological and sports factors, including the child’s age, sex, growth rate, intensity of exercise, frequency, and duration of exercise (2, 3, 5, 6). Therefore, junior athletes, coaches, and sports dietitians must work together to ensure that their players recognize their needs and consume proper sports nutrition. Therefore, young athletes can maximize the benefits of their exercise. Most of those studies focused on the nutritional status of adult athletes rather than the nutritional status of young athletes and adolescents (7). Several nutritionists and sports coaches use the recommendations of sports adults for young and adolescent athletes (3) The dietary requirements of young athletes are greater than the needs of their non-athlete peers of the same age (8, 9). Furthermore, the lack of energy and nutrients for young athletes and adolescents may lead them to not reach the ideal peak bone mass, which may delay their growth and increase their risk of osteoporosis in the future. Additionally, a lack of energy and nutrients in female adolescents can lead to irregular menses and amenorrhea (4).

There is limited information available about the nutritional needs, nutritional intake, wholesome food, and eating disorders for young athletes. Thus, this narrative review uniquely combines research and review papers related to the nutritional status of young athletes aged 5–18 years and details the dietary requirements quantitatively and qualitatively and in terms of wholesome food, food choices, and eating disorders for growing young athletes.

## 2 Materials and methods

A comprehensive literature search was conducted to identify research and review articles pertaining to the relationship between nutritional status and junior high-income athlete performance from 5 to 18 years old. Using the Preferred Reporting Items for Systematic Reviews and Meta-Analysis (PRISMA) guidelines, we employed the following query string in Scopus advanced search: “(junior OR cadet OR children OR adolescent AND NOT adult OR adults) AND athletes AND (sport OR basketball OR football OR runner OR karate) AND (micronutrients OR vitamin OR minerals OR calcium OR iron OR caffeine) AND [macronutrients OR (carbohydrate OR fat OR protein)] AND (supplement OR supplementation)” Additionally, we conducted a manual search of the references from the identified papers, covering the period from July 1980 to May 2024.

Studies that concentrated on adults, non-athletes, diseased individuals, and disabled individuals were excluded. We included only English-language published articles that reported results applicable to this review subject.

### 2.1 Data selection

**Figure 1:**
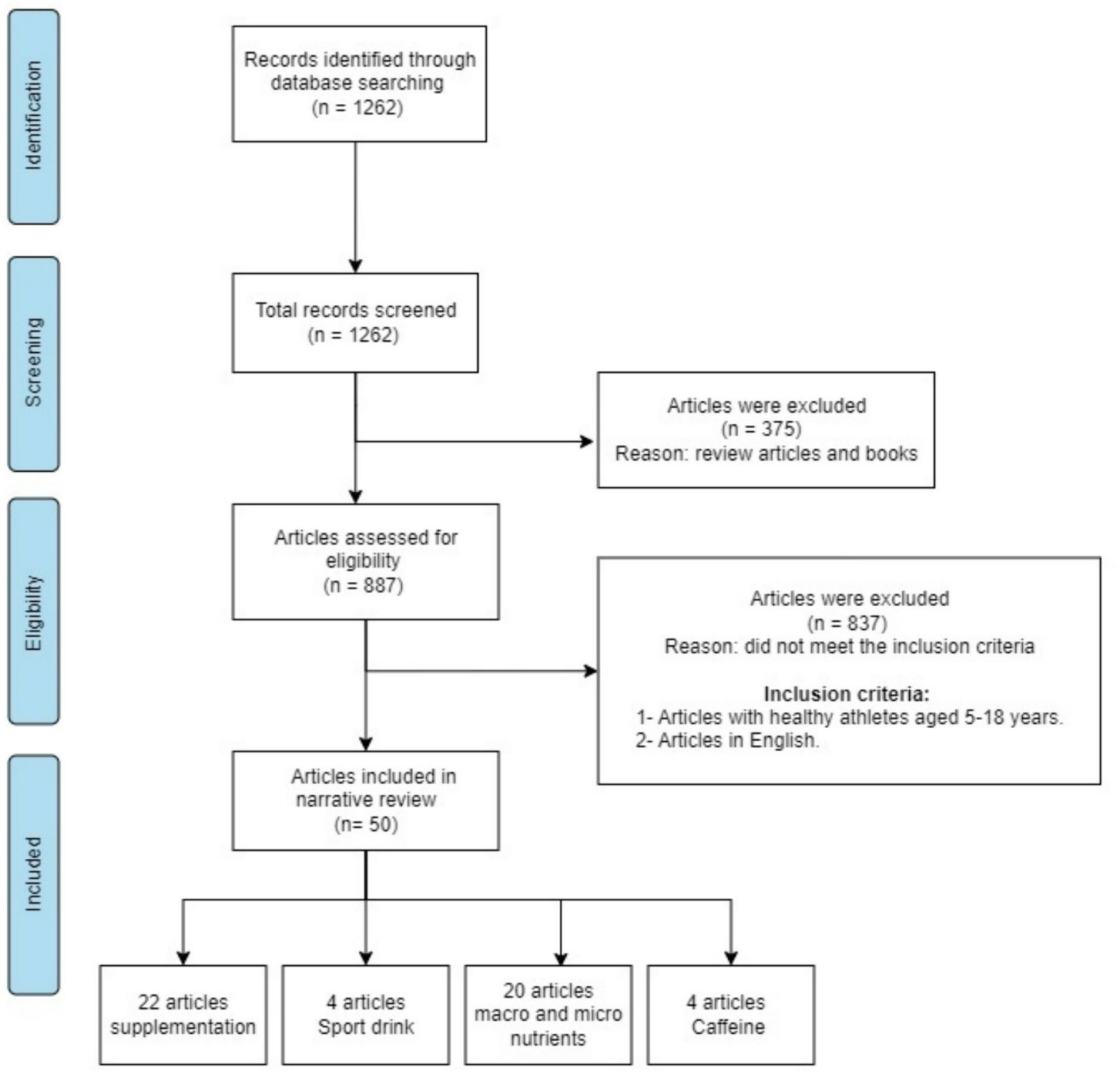
PRISMA diagram with data extraction process and eligibility assessment.

A detailed flowchart of the search strategy can be seen in Figure 1. A preliminary literature search initially identified 1,262 articles, but an initial text analysis for keywords led to the removal of 839 articles from the analysis.

Articles were included based on peer-reviewed published work in the English language and articles with healthy young athletes aged 5–18 years old. However, articles were excluded due to their lack of specification of athlete age. Articles were published in languages other than English, as well as review articles and books were excluded.

### 2.2 Data extraction

The titles and abstracts of studies identified by the search were screened in-dependently and reviewed with the inclusion/exclusion criteria, and only studies related to the nutritional status of junior athletes (≤18 years) were extracted. Data on the effects of macronutrients, micronutrients, supplemental intake, and wholesome food on sports performance in junior higher case athletes were extracted from the eligible studies.

### 2.3 Data synthesis

A narrative synthesis was conducted to collect and summarize the extracted studies. The results of the studies were classified into macronutrients, micronutrients, and supplement intake with sports performance as well as the prevalence of eating disorders among junior athletes. The synthesis included a description of each study’s design, sample size, mean age, sex (male %), intervention strategy, and conclusions.

In this narrative review, all the information from each manuscript was extracted separately by the two authors, who independently extracted the information from each study to reduce the possibility of bias and inaccuracies. Consensus was reached by all the authors on the final list of studies to be included.

## 3 Results

A total of 1,262 research and review articles were obtained in the initial search, and only 48 research articles involving 4,005 juniors related to the nutritional status of junior athletes (≤ 18 years) remained (Figure 1). Table 1 provides a summary of all available research articles on the effects of macronutrients, micronutrients, and supplement intake on the performance of young athletes.

**Table 1:** Comprehensive overview of articles about macronutrients, micronutrients, supplements, and their impact on junior elite athletes’ performance.

NO	Title	Author	Year	Study design	Macronutrient, micronutrient supplement	Sample size	Mean Age ± SD	Sex	Conclusion
								Male%	
1	Muscle glycogen utilization during Rugby match play: effects of pregame carbohydrate.	Bradley W.J. (9)	2016	RCT	CHO	16	18 ± 1	100%	A diet containing approximately 600 g of carbohydrates should be given 36 h before to make sure that all players start the game with the proper levels of muscle glycogen.
2	Dietary analysis of young professional soccer players for 1 week during the competitive season.	Russell M. (10)	2011		CHO, protein, fats	10	17 ± 1.0	100%	Encouraging fiber intake and optimizing the contribution of energy from proteins, fat, and carbohydrates in order to ensure consuming sufficient calories will ultimately boost the preparedness for subsequent exercise.
3	The effect of the Ramadan fast on physical performance and dietary habits in adolescent soccer players.	Meckel Y. (11)	2008		CHO, protein, fats	19	15.1 ± 0.9	100%	Macronutrients such as carbohydrates may relatively contribute and serve as the main nutrient causes for the reduction in physical capacity. The decrease in athletic performance may be significantly contributed to the decreased physical activity.
4	Comparison of site-specific bone mineral densities between endurance runners and sprinters in adolescent women.	Ikedo A. (12)	2016	Cross-sectional	Vitamin D	37	16.1 ± 0.8	0%	Fat-free mass had a significant impact on the variations in bone mineral densities (BMDs) at several sites between sprinters and endurance runners. It appears that there are site-specific correlations between vitamin D intake and BMDs.
5	Physiologic and behavioral indicators of energy deficiency in female adolescent runners with elevated bone turnover.	Barrack M.T. (13)	2010	Cross-sectional	Vitamin D, Calcium	39	15.7 ± 0.2	0%	A lower concentration of serum 25-dihydroxy vitamin D and consuming calcium less than recommended according to the age-specific AI were significantly prevalent among runners with elevated bone turnover EBT.
6	Relationship between dietary factors and bodily iron status among Japanese collegiate elite female rhythmic gymnasts.	Kokubo Y. (14)	2016	Cross-sectional	Iron	60	18.1 ± 0.3	0%	Elite female rhythmic gymnasts are particularly vulnerable to iron deficiency during preseason weight-loss periods due to reduced protein intake. Consuming protein consciously may be essential to preventing the body’s iron deficiency.
7	Dietary intakes and supplement use in preadolescent and adolescent Canadian athletes.	Parnell J.A. (15)	2016	Cross-sectional	Dietary intake of micronutrients and dietary supplements	187	14	45%	Food sources of vitamin D, calcium, iron, and folate (for female athletes) should be the main focus of athlete support staff. Supplementation is generally unnecessary, with the possible exceptions of vitamin D and iron, under physician supervision. To balance depletion against overconsumption, emphasis should be placed on using supplements containing carbohydrates.
8	Multiple micronutrient-fortified rice affects physical performance and plasma vitamin B-12 and homocysteine concentrations of Indian school children.	Thankachan (16)	2012	RCT	Multiple Micronutrient-Fortified Rice	258	8.6 ± 1.8	47%	The fortified rice was efficacious in improving vitamin B-12 status and physical performance in Indian school children.
9	The relationship between food consumption and nutritional status of male junior athletes: a cross sectional study in Sumedang	Insani H.M. (17)	2023	Cross-sectional	Dietary intake of macronutrients and micronutrients	62	16.7 ± 2.4	100%	The findings indicate that the typical nutritional intake among athletes falls short of meeting the nutritional requirements for typical male adolescents. For instance, while the recommended caloric intake for normal male adolescents is 2,650 Kcal, male junior athletes typically consume only 2285.5 Kcal, with only 8.1% meeting the recommended caloric intake. Additionally, athletes generally meet only about 60–80% of the macronutrient and micronutrient needs compared to normal adolescents.
10	Correlation between body image, eating disorders, and nutrient adequacy level with nutritional status of adolescent swimmers in Bogor City, Indonesia; [Hubungan Persepsi Tubuh, Gangguan Makan, dan Tingkat Kecukupan Gizi dengan Status Gizi Atlet Renang Remaja di Kota Bogor, Indonesia].	Assyifa R. (18)	2023	Cross-sectional	Dietary intake of macronutrients and micronutrients	21	14.48 ± 1.63	66.70%	33.3% of adolescent swimmers were identified as at risk of eating disorders, with a higher proportion of women than men falling into this category. A large majority experienced energy insufficiency and did not meet the recommended intake of carbohydrates. Vitamin D fell below the recommended level, while vitamins B1, B2, B3, and vitamin C were consumed adequately.
11	Dietary habits in adolescent male and female handball players: the Swedish Handball Cohort.	Onell C. (19)	2023	Cross-sectional	Dietary intake of macronutrients and micronutrients	1,040	16.6 ± 0.9	51%	The dietary habits of Swedish teenage handball players align reasonably well with sports nutrition guidelines, although they do not fully adhere to the Nordic Nutrition Recommendations (NNR). Among the supplements used, protein/creatine, omega-3 fatty acids, vitamin C, vitamin D, iron, and magnesium were the most prevalent. Micronutrient supplementation was more prevalent among females than males, while protein/creatine supplementation was less common among females.
12	Nutritional profile and oxidative stress in adolescent soccer players.	Guissoni F.M. (20)	2020	Cross-sectional	Dietary intake of macronutrients and micronutrients	20	15–17	100%	The intake of antioxidants and macronutrients is unbalanced among soccer players under the age of 17. The prevention of lipid peroxidation was achieved through the recruitment of dietary antioxidant vitamins. However, the body’s natural defense mechanisms, particularly those related to nutrition, were unable to stop protein oxidation.
13	Nutrition status of young elite female German football players.	Braun H. (21)	2018	cross-sectional	Dietary intake of macronutrients and micronutrients	56	14.8 ± 0.7	0%	A significant proportion of participants did not fulfill the recommended intake levels of protein and carbohydrates and energy balance. Some young female football players were found to have inadequate nutrition, as evidenced by low serum levels of iron and 25(OH)D. To improve fluid and CHO intake and approach energy balance, sports drinks that contain carbohydrates and electrolytes are advised before, during, and after training and games.
14	Comparison of dietary intake, energy adequacy and anthropometric parameters between Indian junior male and female hockey players.	Roy M. (22)	2018	cross-sectional	Dietary intake of macronutrients and micronutrients	40	M: 18.2 ± 2.3	17%	Comparison of dietary intake, energy adequacy, and anthropometric parameters between Indian junior male and female hockey players.
							F: 17.1 ± 2.2		
15	Comparison of food consumption and nutritional statuses of athletic adolescents.	Keser A. (23)	2016	cross-sectional	Dietary intake of macronutrients and micronutrients	138	13.8 ± 1.9	78.20%	The current study’s results, which were based on the current dietary recommendations, showed that most athletes had an inadequate and imbalanced nutritional status because of both overnutrition and undernutrition, as found among some athletes.
16	Nutritional habits among high-performance endurance athletes.	Baranauskas M. (24)	2015	Cross-sectional	Dietary intake of macronutrients and micronutrients	146	17.7 ± 2.9	45%	Eighty percent of Lithuanian endurance athletes did not eat enough carbohydrates with their meals, and 33 % did not get the recommended amount of protein. In fact, an excess of fat causes an excess of cholesterol and saturated fatty acids in the diet of endurance athletes.
17	Nutrition status of junior elite Canadian female soccer athletes.	Gibso J. (25)	2011	Cross-sectional	Dietary intake of macronutrients and micronutrients	33	15.7 ± 0.7	0%	A high proportion of players were not in energy balance, failed to meet carbohydrate and micronutrient recommendations, and presented with depleted iron and vitamin D status. Suboptimal nutrition status may affect soccer performance and physiological growth and development.
18	Effect of nutri-bar in the development of stamina building and exercise-performance in young male-athletes.	Jabeen S. (26)	2021	RCT	Nutri-bars were prepared (110 g) using dates (64 g), dried apricots (16 g), cheddar cheese (8 g), whey protein isolate (12 g) and roasted-chickpea flour (10 g)	12	N\A	100%	The blood serum profile of athletes did not exhibit any noteworthy changes as a result of nutri-bar. However, these bars might be useful to obtain a full effect if the efficacy trials are prolonged to a few months. Additionally, soldiers could use nutri-bars as a meal replacement, in fitness centers, for school-age children, and to fight protein-energy malnutrition (PEM).
19	Caffeine improves various aspects of athletic performance in adolescents independent of their 163 C > A CYP1A2 genotypes.	Spineli H. (27)	2020	RCT	Caffeine	100	15 ± 2	N\A	In adolescent athletes, caffeine enhances both muscular endurance and aerobic performance, independent of the 163 C > A CYP1A2 genotype.
20	The acute effects of caffeine ingestion on reactive agility performance in soccer players.	Egesoy H. (28)	2020	RCT	Caffeine	48	16.5 ± 0.5	100%	Caffeine consumption improves the components of decision time, total agility time, and sprint time. Soccer players’ ability to perform with reactive agility may benefit from caffeine consumption.
21	Caffeine does not augment markers of muscle damage or leukocytosis following resistance exercise.	Machado M. (29)	2010	RCT	Caffeine	15	18.4 ± 0.8	100%	Ingestion of caffeine at 4.5 mg·kg^−1^ did not augment markers of muscle damage or leukocyte levels above that which occurs through resistance exercise alone.
22	Caffeine’s effect on intermittent sprint cycling performance with different rest intervals.	Lee C. (30)	2011	RCT	Caffeine	14	18.7 ± 0.8	100%	In conclusion, caffeine ingestion may be ergolytic, affecting performance and fatigue development in the later stage during a prolonged and intermittent sprint test with a short recovery interval. However, caffeine produces an ergogenic effect in the initial stage of an intermittent sprint performance with a longer recovery interval.
23	Effects of the long-term consumption of hydrogen-rich water on the antioxidant activity and the gut flora in female juvenile soccer players from Suzhou, China.	Sha J.-B. (31)	2018	RCT	Hydrogen-rich water	38	Group 1: 13.7 ± 1.06	27%	Effects of the long-term consumption of hydrogen-rich water on the antioxidant activity and the gut flora in female juvenile soccer players from Suzhou, China.
							Group 2: 12.18 ± 0.86		
24	The effects of the intake of an isotonic sports drink before orienteering competitions on skeletal muscle damage.	Colakoglu F.F. (32)	2016	RCT	Isotonic sports drink	21	Group 1: 15.7 ± 2.2	28%	The effects of the intake of an isotonic sports drink before orienteering competitions on skeletal muscle damage.
							Group 2: 15.4 ± 2.2		
25	Enhancing physical performance in elite junior tennis players with a caffeinated energy drink.	Gallo-Salazar C. (33)	2015	RCT	Caffeinated energy drink	14	16 ± 1	71.43%	In a simulated junior tennis match, the pre-exercise consumption of a caffeinated energy drink containing 3 mg/kg of caffeine increased handgrip force and high intensity running, and it also tended to increase the percentage of points won on service.
26	A caffeinated energy drink improves jump performance in adolescent basketball players.	Abian-Vicen J. (34)	2014	RCT	Caffeinated energy drink	16	14.9 ± 0.8	100%	The intake of a caffeine-containing energy drink (3 mg/kg body weight) increased jump performance although it did not affect basketball shooting precision.
27	Nutritional Supplement Use in a United Kingdom High-Performance Swimming Club.	Newbury J.W. (35)	2023	Cross-sectional	Dietary supplements	44	15 ± 3	87.50%	Both male and female swimmers showed similar overall supplement usage, including both ergogenic and health supplements. However, there were differences in specific supplement types: more males used multivitamins, sports supplements, and protein-enhanced foods, while more females consumed caffeine anhydrous. This indicates a pattern of prevalent supplement use among swimmers from developmental ages (aged 11–14 years) to national-level performance.
28	Prevalence, knowledge and attitudes toward using sports supplements among young athletes.	Jovanov P. (1)	2019	Cross-sectional	Dietary supplements	348	15–18	N\A	The supplementation is common among young athletes (82.2%), 60.6% of whom were male, and is not limited to any particular demographic or activity.
29	Dietary supplement use in young elite athletes and school children aged 11 to 13 years: a cross-sectional study design.	Kratzenstein S. (36)	2016	Cross-sectional	Dietary supplement	562	11.7 ± 0.6	N\A	Young German athletes who are just starting their sports careers exhibit comparable patterns of supplement usage to their peers who are not involved in athletics.Compared to previous research, the observed athletes have a low prevalence of dietary supplement use, which could rise with age and increasing performance level.
30	Analysis of sports supplements consumption in young Spanish elite dinghy sailors.	Caraballo I. (37)	2020	cross-sectional	Dietary supplements	42	12–17	73.80%	In this study, the prevalence of sports supplement consumption among sailors was approximately 50%. Men consume sports supplements primarily for performance-related reasons, but women primarily use them for health and to avoid nutritional deficiencies.
31	Does carbohydrate supplementation enhance tennis match play performance?	Gomes R. (38)	2013	RCT	CHO supplementation	12	18.0 ± 1.0	100%	CHO supplementation does not augment measures of tennis match play performance. However, during prolonged matches or tournaments that require multiple matches in a 24-h time span an athlete may benefit from CHO supplementation.
32	The preliminary analysis of protein catabolism and nitrogen balance in young gymnasts.	Sawicki P. (39)	2018	RCT	CHO and Protein supplementation	5	13 ± 0.5	100%	Immediately after training, supplementing with a combination of carbohydrates and proteins reduced the body’s overall protein catabolism and a specific quantity of nitrogen excreted in the urine. Young gymnasts who are calorie deficient show that poor diet management may have a negative impact on a developing organism’s physical development.
33	Milk protein consumption improves muscle performance and total antioxidant status in young soccer athletes: A randomized controlled trial.	Setiawan M.I. (40)	2020	RCT	Milk protein	20	16_18	100%	Over the course of 28 days, young male soccer athletes who received milk protein supplements saw improvements in their overall antioxidant status and muscle performance.
34	Vitamin C, A and E supplementation decreases the expression of HSPA1A and HSPB1 genes in the leukocytes of young polish figure skaters during a 10-day training camp.	Zychowska M. (41)	2015	RCT	Vitamin C, A and E supplementation	14	14.4 ± 0.3	0%	Antioxidant vitamins A, C, and E supplementation dramatically reduces the expression of HSPA1A and HSPB1 in the leukocytes of young soloist figure skaters who are training hard.
35	Vitamin D supplementation and physical performance in adolescent swimmers.	Dubnov-Raz G. (42)	2015	RCT	Vitamin D supplementation	53	14.1 ± 1.8	61%	No significant differences in performance between participants who became vitamin D sufficient, and those who did not.
36	Iron, hematological parameters and blood plasma lipid profile in vitamin D supplemented and non-supplemented young soccer players subjected to high-intensity interval training.	Jastrzębska M. (43)	2017	RCT	Vitamin D supplementation and vitamin D from sunflower oil	36	17.5 ± 0.6	100%	Little soccer players participating in high-intensity interval training showed no change in hematological or lipid parameters after taking a daily vitamin D supplement.
37	Effect of different dosages of zinc supplementation on nutritional status, aerobic and anaerobic performance in elite female volleyball players.	Eskici G. (44)	2021	RCT	Zinc supplementation	20	15.1 ± 1.07	0%	The zinc supplement is said to have no effect on anaerobic or aerobic performance regardless of dosage; however, because it lowers lactic acid levels, it may lessen fatigue and improve an athlete’s performance.
38	Caffeine supplementation improves countermovement jump in youth soccer players: A pilot study.	Hernández-Camacho J.D. (45)	2017	RCT	Caffeine supplementation	17	17.65 ± 0.48	100%	Consuming 4 mg of caffeine per kilogram of body mass may serve as an ergogenic aid to enhance soccer performance.
39	The effects of acute caffeine ingestion on decision-making and pass accuracy in young soccer players: A preliminary randomized controlled trial.	Jafari N. (46)	2024	RCT	Caffeine supplementation	12	16–17	100%	The consumption of 3 mg of caffeine per kilogram of body weight immediately did not lead to statistically significant changes in the decision-making skills or pass accuracy of young football players. However, a majority of participants exhibited lower scores in decision-making and the Loughborough Soccer Passing Test (LSPT) following caffeine consumption.
40	12-week curcumin supplementation may relieve postexercise muscle fatigue in adolescent athletes.	Bai KY (47)	2023	RCT	Curcumin supplementation	28	17 ± 1	75%	Regular curcumin supplementation during exercise can relieve the associated muscle soreness and fatigue. Although the effect of curcumin supplementation on exercise performance may vary from person to person, it could be considered to cooperate into nutritional supplements in regular training in adolescent athletes.
41	The potential of Curcuma extract to alleviate muscle damage in amateur soccer players.	Rosidi A. (48)	2022	RCT	Curcuma extract supplementation in ice cream	20	14–18	100%	Curcumin extract ice cream at a concentration of 250 mg per 100 g has the potential to mitigate muscle damage in soccer players following a rigorous 21-day training regimen. The use of Curcumin extract ice cream may help dampen the inflammatory response post-exercise.
42	Effect of glutathione supplementation on swimmers’ performance.	Petrov L. (49)	2021	RCT	Glutathione supplementation	24	18.7 ± 3.78	58.33%	Glutathione supplementation at a dose of 250 mg daily for six weeks enhances elite swimmers’ ability to adjust to training regimens.
43	Effect of spirulina maxima on postprandial lipemia in young runners: a preliminary report.	Torres-Durán PV (50)	2012	RCT	Spirulina maxima	41	15.7 ± 4.5	51.20%	Spirulina diminishes postprandial lipemia after a high-fat meal in young athletes, especially between 10 and 16 years of age. We observed this effect since 1.5 h, although at 3–4.5 h, the decrease is higher. This effect was observed despite a short detraining period.
44	Changes in maximal strength and body composition after different methods of developing muscle strength and supplementation with creatine, L-carnitine and HMB.	Kruszewski M. (51)	2011		Creatine, L-creatine and β-hydroxy-β-methylbutyrate (HMB)	182	N\A	100%	Significant differences in the examined parameters were also detected within the group of advanced powerlifters practicing weightlifting between those who were supplemented with creatine and those who were given placebo. Thus, the use of creatine in the development of physical capacity in advanced athletes may be advisable.
45	Effect of low dose, short-term creatine supplementation on muscle power output in elite youth soccer players.	Yáñez-Silva A. (52)	2017	RCT	Creatine monohydrate supplementation	19	17.0 ± 0.5	100%	Giving elite young soccer players a short-term, low-dose oral Cr supplementation improved their muscle power output.
46	Beta-alanine supplementation enhances judo-related performance in highly trained athletes.	De Andrade Kratz C. (53)	2017	RCT	Beta-alanine supplementation	23	17 ± 2	100%	In well-trained judo athletes, 4 weeks of beta-alanine supplementation significantly enhanced judo-related performance, indicating that this modality can also profit from beta-alanine’s ergogenic effects.
47	Effects of acute supplementation of L-arginine and nitrate on endurance and sprint performance in elite athletes.	Sandbakk S.B. (54)	2015	RCT	L-arginine and nitrate supplementation	9	18 ± 0	100%	In cross-country skiers with endurance training, L-arginine and nitrate supplementation had no effect on exercise economy or endurance running performance.
48	Dietary nitrate supplementation improves rowing performance in well-trained rowers.	Bond H. (55)	2012	RCT	Nitrate supplementation	14	16.7 ± 0.5	100%	Maximal rowing-ergometer repetitions improved after nitrate supplementation in the form of 500 mL of beetroot juice for 6 days, especially in the later phases of exercise.

Several studies have shown that the majority of young athletes do not adhere to healthy nutrition guidelines and suffer from energy imbalances (19, 20, 23, 25), and are more susceptible to eating disorders (18). In addition, the typical dietary intake did not meet the recommended intake to meet their needs for macro- and micronutrients (17, 21, 22, 24) (Table 1).

### 3.1 Carbohydrates

Carbohydrates (CHOs) are the main source of energy for junior athletes’ muscles and the exclusive fuel for the brain and nervous system. The CHO is a fundamental component of providing young athletes with energy for growth and development. Moreover, it is crucial to improve cognitive function, maintain blood glucose levels, and replenish glycogen stores in muscle and liver (56). Several studies have indicated that aging is inversely related to athletic endurance, as the level of glycogen stores is greater in junior athletes than in elderly individuals (2). Muscle glycogen is the most readily available fuel for exercise, and its depletion leads to early fatigue and poor performance, as mentioned by Purcell et al. (57). Muscle glycogen helps young athletes adapt to and perform endurance exercises. As the intensity of exercise increases, more muscle glycogen is used. However, as the duration of exercise increases, the energy used shifts to blood glucose. Studies have shown that the depletion of muscle glycogen occurs faster in young athletes than in adult athletes (58, 59).

Studies have shown that exact CHO requirements cannot be determined because they vary according to exercise level, age, and sex. Moreover, CHO should account for 45–65% of total caloric intake (60, 61). However, the recommended dietary allowance (RDA) for consuming CHO among juniors is 130 g/day, based on the amount that the brain needs to function properly (62). A study of the effect of pre-match carbohydrates on professional players with an average age of 18 years showed that a diet of approximately 600 g of carbohydrate should be given 36 h before all players start the game with proper levels of muscle glycogen (9). Table 1 shows the results of several studies on the importance of CHO in the diet of young athletes (9–11, 39).

There is no doubt that these macronutrients, especially CHO, are extremely important in a healthy diet and are crucial points for optimal athletic performance (63). Studies have indicated that the quality and quantity of the meal as well as meal timing have a significant impact on the player’s performance and degree of endurance while practicing sports (2, 64). Studies have emphasized that young athletes should consume CHO from good and healthy sources, including whole grains, fruits, vegetables, milk, yogurt, and honey rather than refined sugars. Additionally, the consumption of high-glycemic index foods within 30 min to 2 h immediately after exercise accelerates recovery and restores depleted glycogen stores. Studies have shown that foods containing fructose (fruits) and galactose (milk and dairy products) replenish glycogen stores faster than glucose content (2, 64, 65).

### 3.2 Proteins

Protein is an essential macronutrient for junior athletes because it plays a crucial role in their growth, development, overall athletic performance, and enhancement of exercise training (56). Protein is needed to build junior muscle, repair, recover, and meet increased energy demands, as it is a vital nutrient for active populations (66). During peak growth, increases in lean body mass can reach approximately 2.3 and 3.8 g/day in females and males, respectively, which is a threefold increase from the prepubertal period. Considering that total energy intake is essential in the assessment of protein requirements because of suboptimal energy intake, in addition to using liver glycogen, endogenous proteins are also used to maintain blood glucose homeostasis, thus reducing the availability of proteins for primary functions (56). Athletes have higher requirements for dietary protein intake than their sedentary peers (7).

The general recommendations for daily protein intake ranged between 0.8 and 1.2 g of protein per kg of body mass. In comparison, both the American College of Sports Medicine and the American Dietetic Association recommend protein intake between 1.2 and 1.8 g/kg body mass for active adults, which is a sufficient requirement for junior athletes (7, 67, 68). On the other hand, the International Society of Sports Nutrition recommends consuming approximately 1.4–2 g per kg of body mass of protein per day for most exercising individuals to maximize training-induced adaptations (69). The recommended dietary intake (RDA) for young males and females is 0.95 g.kg^−1^. day^−1^ from 4 to 13 years of age and 0.85 g.kg^−1^/day from 14 to 18 years of age (70). Young male and female athletes had slightly higher recommended protein intakes of 1.4–2 g.kg^−1^. day^−1^ (2). In an older study from 2004, the amount of protein recommended for adolescent athletic boys aged 15–18 years was 0.9 g.kg^−1^. day^−1^, whereas that of adolescent athletic girls of the same age was slightly less than 0.8 g.kg^−1^. day^−1^. The European Union and the United Nations recommend two to three times the recommended protein intake (71). Elevated protein intake can be achieved normally for different youth age groups through regular dietary patterns (7). As the benefits of integrating high-protein food after exercise are well described, there is a large increase in the use of protein-based supplements in market stores as well as online stores (67, 72).

Additionally, nutritional supplements, particularly proteins, are prevalent among athletes at all levels, regardless of their necessity of consuming an adequately balanced diet (73). In conjunction, Baltazar-Martins and colleagues studied dietary supplements among 527 high-performance athletes. One hundred and eleven were junior athletes aged <15–20 years; 30% of them stated that they had used supplements, and the most prevalent dietary supplement consumed was protein, which accounted for 40% of the sample (74). Additionally, among 574 Japanese track and field athletes, including 275 junior athletes aged less than 20 years, the prevalence of consuming nutritional supplements among junior athletes was 58.9%, and the most widespread components were amino acids (49.3%), vitamins (48.3%), minerals (22.8%), and proteins (17.8%) (73). However, the inclusion of foreign components in protein supplements represents a risk not only to athlete health but also to their ability to follow the anti-doping code imposed by the World Anti-Doping Agency (WADA) (39, 72). Consequently, some athletes have been documented to test positive for WADA-related substances in a variety of sports globally (72). Thus, according to a pilot study performed by Whitehouse and Lawlis (72), 60% of the junior athletes in the present study reported using protein supplements.

Additionally, they suggest that adolescent athletes be at risk of consuming such products, as 19% of them obtain the information from the internet and 17% buy their supplements online (72). On the other hand, increased energy requirements are likely caused by increased food and nutrient intake; however, athletes may unintentionally fail to adjust their diets to increase energy expenditure, impacting overall diet quality (75, 76). Table 1 shows the results of several studies on the importance of protein in the diet of young athletes (10, 11). Furthermore, the ability of young team sports players to achieve good nutritional practices has been poorly addressed, and there has been little research regarding the nutritional needs of young elite athletes, with recommendations typically based on adult values (75).

### 3.3 Fats

Fat is essential for all athletes, including junior athletes (57). The caloric needs are increased in people who exercise; therefore, the dietary lipid content may be greater (7). Young athletes may be able to utilize slightly more fat than sedentary people (8), and younger children use a greater percentage of fat because of their adaptive response to support their increased requirements, growth demands and increased energy expenditure during exercise (7, 8, 77). The diet should contain 25 to 35% of the total energy needs of junior athletes according to the acceptable macronutrient distribution range (AMDR) (78); this aligns with dietary recommendations for athletes and non-athletes and remains consistent with current guidelines and public health (56, 79). Fat is a necessary ingredient of any healthy and balanced diet because it is included in biological body processes and serves as an energy source, as mentioned in previous studies (10, 62). Furthermore, fat is a crucial energy source during exercise at low and moderate intensities (7, 77). Additionally, PUFA (n-6 and n-3) families are diet-based and are considered critical for brain development (80). Fat is essential for fat-soluble vitamin absorption (8); some fat-soluble vitamins, such as vitamin D and K, also contribute to proper bone growth, which is necessary for junior athletes and performance and prevent fat accumulation; as well, vitamins A, E, and D improve strength, performance and muscle recovery (81, 82). Approximately 50% of adult body and bone mass is achieved during adulthood. Significant changes in lean mass and fat tissue are also observed during the transition from childhood to adulthood (7), and during this period, dietary fat, such as sex hormones, is essential for facilitating hormone synthesis (7).

Fat contributes to normal body function and healthy growth maturation in young athletes (7, 56). A study mentioned that high-fat diets appear to maintain testosterone blood concentrations better than low-fat diets (79). Testosterone is an anabolic hormone that increases muscle mass and is essential for improving strength, power, and endurance (83, 84). The pre workout meal should be low in fat to minimize gastric upsets, which reduce performance (85, 86). Eating high-fat meals before a workup can reduce the level of growth hormone secretion by 40% during exercise, which impairs muscle adaptation and growth (68). Table 1 shows that a study of juniors of soccer players concluded that encouraging fat intake and optimizing the contribution of energy from fat to ensure sufficient caloric intake will ultimately boost exercise performance (10). In adults, it has been found that low-carb, high-fat diets can also lead to decreased exercise performance (87). However, excessive lipid intake can also lead to postprandial oxidative stress, which is associated with impaired vascular and metabolic function (7).

### 3.4 Vitamins

Vitamins are essential organic compounds that play crucial roles in physiological and biochemical functions throughout the body and are relevant to athlete performance (88, 89). Essential vitamins cannot be synthesized in the body, either at all or in insufficient amounts, and many of them are essential in various processes. Others are of crucial importance for the development of the compounds required by the body. For example, vitamin D has hormone-like functions, while vitamin A acts as a cell regulator of tissue growth and differentiation, and vitamins C and E act as antioxidants (89).

Research on the effects of vitamins and vitamin supplements on junior athletes is limited and inconclusive. In this review, we found only studies on vitamin D and its effect on junior high-income athletes. The effects of other vitamins on junior athletes were not the topic of any scientific studies (Table 1). Vitamin D status is critical not only for preventing nutritional rickets but also for reducing bone mineral density, fracture risk, and risk of deficiency in young athletes, especially for those less exposed to sunlight (90). In a comparative study of site-specific bone mineral densities between endurance runners and spinters in adolescent women, site-specific associations were found between bone mineral density and vitamin D intake (12) (Table 1).

Young athletes who consume insufficient calories or whose diet lacks rich sources of daily vitamin D may require vitamin D supplements (91). This explains the widespread interest in calcium- and vitamin D-fortified food, particularly among individuals who lack sufficient exposure to sunlight (7). Table 1 presents the findings of a study conducted on female junior soccer athletes. The study concluded that low levels of serum hydroxyvitamin D and inadequate calcium and vitamin D consumption requires more precise assessments of bone formation, such as dual-energy X-ray absorptiometry (DXA) (25). Another study concluded that adolescent female runners with elevated bone turnover (EBT), who consumed less calcium and 25-dihydroxy vitamin D than recommended according to the age-specific AI, were exposed to amenorrhea and a decrease in body mass (13) (Table 1).

### 3.5 Minerals

In addition to their relative paucity in the body and diet, minerals are key regulators of function and health, such as work performance (92). Minerals also have biochemical functions that potentially affect physical performance (92). Calcium is critical for enzyme activity, bone health, and muscle contraction (13). Poor calcium intake, such as dual energy X-ray absorptiometry also results in robust measurements of bone development (25).

Two studies revealed that consuming less calcium than recommended according to the age-specific AI was significantly more prevalent among runners with elevated bone turnover (EBT) and warranted more accurate measurements of bone development among athletes with a mean age of 15.7 years (13, 25). Kokubo et al. (14) assumed that Japanese college elite female rhythmic gymnasts are particularly vulnerable to iron deficiency during preseason weight loss periods due to reduced protein intake, which highlights the importance of protein intake for enhancing bodily iron status. A study by Gibson et al. (25) revealed that a greater proportion of athletes had poor serum iron storage and had lower intake levels than did those with DRI micronutrient recommendations or sports nutrition. Moreover, physiological growth, energy metabolism, immune function and soccer performance may be affected by suboptimal nutritional status among a sample of Canadian female soccer athletes with a mean age of 15.7 years (25).

### 3.6 Consumption of multiple micronutrient-fortified rice

“Hidden hunger” is an expression that indicates deficiencies of vital micronutrients, vitamins, and minerals, such as iron, zinc, vitamin A, and B vitamins, which are common among populations in developing countries (93). This universal issue usually arises from high intakes of cereals and low intakes of fresh fruits, vegetables, and animal products. Thus, fortifying stable foods may be a promising way to counteract nutrient deficiencies in limited-source countries (94). In a randomized, double-blind, controlled study conducted on Indian schoolchildren aged between 6 and 12 years old, the administration of multiple micronutrient-fortified rice 6 days/week for 6 months, as a way to encounter micronutrient deficiencies in the country, meaningfully improved their plasma vitamin B-12 and homocysteine levels, as well as their physical performance (16) (Table 1).

### 3.7 Supplements

Nutritional supplements are products that come in numerous states and in various forms, such as liquids, powders, soft gels, gelcaps, tablets, and capsules; are targeted to supply and support the diet; and include vitamins, minerals, botanicals, and amino acids (95). The dietary requirements of young athletes are greater than those of non-athletics. Additionally, increased requirements for high-intensity exercise increase the likelihood of nutrient deficiency (15). For children, depending on the type of exercise, the BMR might be 2–4 times greater for BMRs than for non-athletes (39). Therefore, in young athletes, nutrients, such as CHO, may be susceptible to deficiency, especially during activity; magnesium, zinc, iron, calcium, vitamin E, and vitamin D (15).

A recent study of nutritional supplement use for swimmers aged 11–14 years in the United Kingdom showed similar use for males and females. There were differences in specific supplement types: more males used multivitamins, sports supplements, and protein-enhanced foods, while more females consumed caffeine anhydrous, according to the results of the study, which included 44 highly trained swimmers on a national talent pathway, 87.5% of whom were male (35) (Table 1).

Another study showed the widespread consumption of nutritional supplements among young athletes, which is not limited to any particular demographic or activity (1).

However, another study revealed that young men and women take nutritional supplements for reasons related to performance for males and for reasons related to health and to avoid nutritional deficiency for females (37).

#### 3.7.1 Protein supplementation

Concerning recovery from exercise, the ingestion of protein supplements can enhance skeletal muscle protein synthesis by stimulating a greater ratio of myofibrils to mitochondria (96). Despite the fact that athletes require extra protein being widely proven, the majority of athletes already consume a balanced diet that covers their protein needs (97). Moreover, there is insufficient evidence to support the belief that consuming additional protein or amino acids leads to enhanced athletic performance (97).

In contrast, Table 1 shows that supplementing young gymnasts with a combination of CHO and proteins immediately after training reduced the body’s overall protein catabolism and the specific quantity of nitrogen excreted in the urine (39). Additionally, a study of junior Canadian athletes recommended the use of supplements involving CHO to balance deficiency against overdosing supplements, as clearly reported by the conclusion of the study (15, 53) (Table 1).

#### 3.7.2 Carbohydrate supplementation

Carbohydrate (CHO) supplementation is normally suggested during exercise to augment endurance exercise performance as it fuels the skeletal muscles and the central nervous system (62, 98). Conversely, in several studies showing the importance of CHO administration to athletes during exercising or before starting matches, it was found that the consumption of a standardized CHO solution (Maltodextrin solution; 1 g/kg^−1^; 10%) did not improve the tennis match play performance in 180 min. However, CHO supplementation may be beneficial in prolonged matches with multiple bouts within a 24-h timeframe (38) (Table 1).

#### 3.7.3 Vitamin and mineral supplementation

Dietary supplements are mostly unnecessary, considering that it is possible to administer vitamin D and iron supplements under the supervision of a doctor, as mentioned in Table 1 (15).

Table 1 shows that a study of junior Canadian athletes concluded that dietary sources of vitamin D, calcium, iron, and folate (for female athletes) should be the basic concern and interest (39). A study of junior Canadian athletes revealed that 100% of participants consumed some supplements, protein powders, sport bars, multivitamins, multiminerals, vitamin C, and vitamin D, which are the most regularly used supplements (15). Another study in 2023 mentioned that some athletes may benefit from multivitamin supplements if they are incapable of meeting their micronutrient requirements due to deficiencies or absorption defects (52).

A cross-sectional study revealed that junior elite German athletes aged 11–13, who recently embarked on their sports journey, consumed lower amounts of dietary supplements when compared to their non-athletic peers, with a percentage of 14 and 20%, respectively. It is thought that this percentage among athletes is suspected to rise with age and performance level. Calcium, magnesium, vitamin C, and multivitamin supplements were the most frequently consumed minerals and vitamins between them (36).

HSPA1A and HSPB1 are considered indicative of stress and the breakdown of damaged proteins, consequently, their expression is frequently monitored during exercise. Thus, a study investigated the effect of supplementation of antioxidant vitamins A (16 μg/kg/day), vitamin C (8 mg/kg/day), and vitamin E (1 mg/kg/day) on the expression of these genes in the leukocytes of young soloist figure skaters who endure concentrated training regimen. It was found that Vitamin supplementation resulted in a 40 and 25% decrease in HSPA1A and HSPB1 expression among the skater’s athletes, thereby promoting overall health and improving the training outcomes (41) (Table 1).

Considering the importance of zinc intake through the athlete’s diet on performance, since it has a pivotal role in the activation of numerous enzymes involved in energy metabolism (99) and is important in reducing the levels of oxidative stress in skeletal muscles and throughout the body (100) a study was done to evaluate its effect on nutritional status, aerobic, anaerobic performance and fatigue among elite female volleyball players, revealed that intake of two different doses of zinc sulfate (220 and 440 mg/day) not only decreased the levels of plasma lactate after being elevated during exercise but also led to a notable improvement in daily energy, protein, and fat intake (44) (Table 1).

#### 3.7.4 Glutathione supplementation

Glutathione (GSH), the most prominent non-protein thiol in the cells, plays a significant role in redox homeostasis, detoxification, and iron metabolism in bacteria, plants, and human beings. GSH acts by directly interacting with foreign substances and neutralizing reactive oxygen species (ROS) in the body (101). GSH levels decline under oxidative stress, while GSSG levels increase in their oxidized form. These changes in the concentrations of GSH and GSSG in the blood were also observed when athletes trained hard during physical exercise (102, 103) it was found that supplementation with GSH positively affected aerobic metabolism in skeletal muscles, leading to reduced exercise-induced muscle fatigue (104). According to a recent study, supplementation of 250 mg/day of glutathione for 42 days resulted in a progressive decline of cortisol and cortisone urine values of elite swimmers of the Bulgarian national swimming team; it also enhanced the swimmer’s performance and adjustability to training regimens (49) (Table 1).

#### 3.7.5 Nutri-bars supplementation

Nutri-bars were primarily brought to the market for soldiers and athletes to provide them with an immediate source of nutrients and energy (105). Nutri-bars may aid in an athlete’s muscle growth, improve workout performance, and protect against injury. Consuming 2 Nutri-bars/day consisting mainly of dates, dry apricots, whey protein isolates, roasted chickpea flour, and cheddar cheese for 30 days led to substantial development of young male athletes’s stamina-building, performance, and weight management. However, no significant changes in the serum blood profile of the athletes were noticed, which was attributed to the brief term of the study (26) (Table 1).

#### 3.7.6 Hydrogen-rich water supplementation

Recently, hydrogen has been acknowledged as an innovative selective antioxidant with potential valuable applications in sports (31). Thereafter, sports science scientists captured growing attention toward hydrogen-positive anti-oxidant, anti-inflammatory, and anti-apoptotic effects in addition to its role in the regulation of body alkalinity (106). The positive effects of dissolving hydrogen in water have been steadily established in both human and animal trials. In a study for 60 days, drinking 1.5–2 L per day of hydrogen-rich water exhibited meaningful anti-inflammatory and antioxidant properties by diminishing levels of serum malondialdehyde, interleukin-1, interleukin-6, and tumor necrosis factor-α, while remarkably increasing serum superoxide dismutase, total antioxidant capacity, and hemoglobin concentrations in whole blood. Moreover, the intake of hydrogen-rich water improved the diversity and richness of gut flora among young Chinese football players (31).

#### 3.7.7 Creatine

Creatine is a nitrogen-based compound that is present in skeletal muscle (97). When needed, the body uses the phosphocreatine (PCr) as the first support source of adenosine triphosphate (ATP) in skeletal muscle during high-intensity anaerobic exercise (97).

Oral creatine monohydrate (Cr) supplementation is used to increase creatine muscle storage and increase high-intensity exercise bouts (52). Most of the energy necessary to resynthesize ATP while practicing short-term, high-intensity exercise bouts is provided by a mixture of PCr analysis and anaerobic glycolysis. However, when PCr decreases, the performance decreases because ATP cannot be recycled at the ratio required (52). Giving elite young soccer players short-term, low-dose oral Cr supplementation improved their muscle power output, as reported in a previous study (52) (Table 1).

Another study’s findings indicated that significant differences in the examined parameters and substantial disparities in maximal strength were detected among bodybuilders and powerlifters involved in weightlifting who were given supplementation of creatine and L-carnitine, from those receiving a placebo. Accordingly, combining creatine administration with the training regimen of advanced athletes may be recommended to enhance their physical capabilities (51) (Table 1).

#### 3.7.8 Beta-alanine

Along with histidine, beta-alanine is a nonessential amino acid that contributes to the synthesis of the dipeptide carnosine, but the availability of beta-alanine is the major key factor for the formation of carnosine in skeletal muscle (53). Carnosine is used as a buffering agent by receiving hydrogen ions (H+) and has an essential role in maintaining intracellular pH homeostasis, thus increasing tolerance and delaying muscle fatigue during continuous anaerobic exercise (107). It has been found that long-term beta-alanine supplementation (usually 4–10 weeks) may enhance performance in different types of sports by reducing acidosis in muscles, particularly those sports that require glycolytic demand (53). In well-trained judo athletes, 4 weeks of beta-alanine supplementation significantly enhanced judo-related performance, indicating that this modality can also benefit from the ergogenic effects of beta-alanine, as clearly reported in a previous study (53) (Table 1).

#### 3.7.9 Nitric oxide

Nitric oxide (NO) is a gas-based molecule (108). Nitric oxide is essential for the regulation of numerous body processes, including muscle contraction, body metabolism, neuronal efficiency, and immunity (54). Increased NO availability may increase oxygen and nutrient supplies to muscles and improve performance and recovery during endurance training (54). NO is generated through separate pathways that depend on precursors involving L-arginine and nitrates, with L-citrulline acting as a powerful precursor of L-arginine (108). Most notably, NO plays a crucial role in endothelial function, enhancing the relaxation of vascular smooth muscle and subsequent vasodilation, which enhances mitochondrial effectiveness, muscle contraction, and aerobic and anaerobic metabolism (108).

In cross-country skiers with endurance training, L-arginine and nitrate supplementation had no effect on exercise economy or endurance running performance in junior athletes, as clearly reported in a previous study (54) (Table 1). Table 1 presents a study on junior rowers in which maximal rowing-ergometer repetitions improved after nitrate supplementation in the form of 500 mL of beetroot juice for 6 days, especially in the later phases of exercise (55).

#### 3.7.10 Spirulina maxima supplementation

One of the greatest benefits of spirulina for the athletic body is that it improves muscle strength and increases the individual’s endurance, as oxidative damage resulting from exercise is a major cause of muscle fatigue. Spirulina contains antioxidant properties that can help athletes and physically active individuals reduce this damage (109, 110).

The consumption of highly nutritive Spirulina maxima, a consumable microorganism, was found to lower postprandial lipemia following a high-fat meal in young athletes, particularly those aged 10 to 16. The impact has been detected for 1.5 h, with a more noticeable decline around 3–4.5 h after ingestion of spirulina (50) (Table 1).

#### 3.7.11 Curcumin supplementation

Curcumin has positive effects for athletes and physical exercises, including delayed-onset muscle soreness (DOMS), as well as reducing inflammation and oxidative stress (111).

In a study conducted on 28 middle and high school athletes (21 boys and seven girls) with a mean age of 17 years. A noteworthy reduction in the 8-OHdG level, muscle fatigue score, and muscle soreness score, along with an increase in BMR and fat-free mass, was observed in athletes who were supplemented with curcumin for 12 weeks when compared to the control group (47) (Table 1).

In another study, turmeric extract was added to ice cream to study its effect on muscle damage and signs of inflammation in amateur soccer players. It was found that curcumin extract ice cream at a concentration of 250 mg per 100 g has the potential to mitigate muscle damage in soccer players following a rigorous 21-day training regimen. The use of curcumin extract in ice cream may help dampen the inflammatory response post-exercise (48) (Table 1).

#### 3.7.12 Milk protein supplementation

Milk protein supplements are often used by athletes and bodybuilders with the aim of improving athletic performance and increasing the size of muscle mass because they contain valuable amino acids that are similar to the amino acids found in skeletal muscles (112).

For example, a randomized control study conducted on 20 males, aged 16–18-year-old soccer athletes indicated that 4 weeks of milk protein supplementation resulted in increased muscle mass, which in turn improved the muscle performance of the athletes, particularly due to the whey protein content of milk proteins (40) (Table 1).

#### 3.7.13 Caffeine and caffeine supplementation

It has been proven that caffeine is a substance that helps you exercise at a higher intensity and for a longer time in both anaerobic and aerobic performance (113). Therefore, several supplements intended for the pre-workout supplements and fat burners include caffeine (61, 113). Although coffee is considered the main source of caffeine in diets, very few studies have tested the effect of caffeine on the athletic performance of young people.

Table 1 shows four studies of the effect of coffee on the athletic performance of young people **≤**18 years. These studies concluded that caffeine enhances the intensity and duration of sports activity and also reduces the feeling of pain and fatigue during exercise (27–30). It is also likely that caffeine consumption at a dose of 4.5 mg/kg body mass does not cause signs of muscle damage or white blood cell levels higher than those that occur during resistance exercise (29).

Several recent studies have indicated that caffeine supplements increase an athlete’s physical activity, muscle endurance, and muscle pain (114, 115).

It is worth noting that caution should be exercised when using caffeine supplementation for females, as the majority of studies included only male participants (115). Further, in a systematic review of the effect of caffeine supplementation on athletic performance depending on sex, it was concluded that the ergogenic effect of acute caffeine intake on anaerobic performance might be higher in men than in women (116).

As part of the ongoing efforts undertaken by researchers to study the effect of caffeine on the brain and the body in general, a recent study of the effect of caffeine intake on the decision-making process and accuracy of exercises for young football players aged 16–17 years found that consuming caffeine (3 mg/kg body weight) had no effect on decision-making or accuracy of passes, according to the results of the study, which included 12 male volunteers (46) (Table 1). On the other hand, an experimental study of young football players aged 17 years concluded that consumption of caffeine supplementation (4 mg/kg body weight) enhances the jumping skills of football players (45).

#### 3.7.14 Caffeinated energy drink

Sports drinks are electrolyte drinks. Its main purpose is to restore water and electrolytes lost during intense exercise and sweating. There are also energy drinks that are often confused with sports drinks and usually contain large and varied amounts of caffeine. These drinks can enhance athletic performance in the short term (117, 118). There must be a balance between the amount of beverages consumed and the net volume of sweat lost in order to make allowance for ongoing obligatory urine loss (118).

Table 1 shows two studies on the effect of consuming caffeinated energy drink on the athletic performance of 16-year-old youth. It was shown that consuming caffeinated energy drink (3 mg/kg body weight) led to an increase in the athletic performance of basketball and tennis players.

There are also sports drinks that are often confused with energy drinks (119). Colakoglu et al., found that drinking isotonic, supportive, sports drinks pre-exercising reduced the extent of skeletal muscle injury as it drops myoglobin and creatinine kinase serum levels (32).

### 3.8 Wholesome foods and food choices

Both food and sports are expressed as “medicine,” and the application of their continuous interrelationship has a huge impact, especially on child and adolescent athletes, considering it a simple tool for improving athletes’ health (57).

In terms of junior elite athletes, nutrition is not solely essential to fulfill their increased energy needs for training but also to address the higher demands required for their growth and development during puberty (57). Unfortunately, most of the research conducted on junior elite athletes concentrated on single-nutrient analysis as a nutrient-based approach (120, 121) rather than focusing on the food-based approach, which gives a better insight into diet quality and the clarification of diet health relations in terms of whole dietary patterns such as the variety, frequency, and quantity of food choices (122, 123).

In fact, despite the expanding wealth of scientific data on the influence of sports nutrition on performance as well as the presumed ease of access to credible information; it has been revealed that athletes are more prone to exhibit or develop eating disorders, having a greater risk than other inactive individuals (124). This is because of the correlation between being thin and enhanced performances in sports where appearances or weight categories are relevant. Furthermore, many studies indicate that athletes still lack the knack to integrate nutritional recommendations into optimally balanced diets with diverse, high-quality, and proper serving sizes of food choices (124).

Monitoring young athletes’ eating patterns is fundamental since their attitudes regarding meals and dietary practices during this period depend on multiple factors, including age, history with food, flavor preferences, nutritional knowledge, beliefs, geographical location, and cultural context. This in turn can affect their health, training, and performance, as well as their optimal growth and development (123, 125). Typically, a lack of consumption of nutrient-dense foods, particularly vegetables and fruits, is combined with an overindulgence of protein and low-nutrient options, such as alcoholic beverages and soft drinks (120). In addition to intakes below the DRI of several micronutrients are noted among young athletes (126). Sports bars, drinks, and gels are some examples of commercial food items particularly tailored for consumption by athletes, providing specific nutrients such as carbohydrates, fluids, and electrolytes to improve their performance in both training and competitions, besides providing high quantities of proteins that aid in training adaptation and recuperation (127). On the other hand, when compared to traditional wholesome foods full of nutrients and phytochemicals, sports foods contain limited numbers of specific nutrients, which, when over reliant on, can result in inadequate nutrient intake and a restricted range of food choices. So their role should not be exaggerated, as they cannot serve as a surrogate for an athlete’s entire diet. Indeed, the goals of using sports foods can be greatly achieved by the selection of wholesome, everyday functional foods (127).

In general, dairy products, eggs, meats, seafood, beans, fruits, vegetables, whole grains, honey, and nuts are some kinds of wholesome foods with functional properties that have been acknowledged for their physiological effects on young athletes’ performance, bone health, immune system, antioxidant, antibacterial, and inflammatory responses (128–131). As for junior elite athletes, according to Setiawan et al. (40), a shortage of both nutrition knowledge and food choices was found in young athletes of a mean age of 16, which affected their intake of grain products, vegetables, and dairy products, which are significant to meet their high demands of carbohydrate and micro-nutrient needs and to ensure optimum growth and performance.

### 3.9 Risk factors and prevalence of eating disorders among junior athletes

According to the Diagnostic and Statistical Manual of Mental Disorders (DSM-5) and the International Classification of Diseases (ICD-11), anorexia nervosa (AN), bulimia nervosa (BN), restrictive eating disorder, and binge-eating disorder (BED) are some classifications of what are known as eating disorders (EDs) (132–134). Eating disorders (EDs) are severely entrenched psychiatric illnesses, considered a global health issue with significant morbidity and diverse symptoms, responsible for most cases of deaths among mental illnesses (135, 136).

Characterized by disturbed eating (DE) patterns, negative body image perception, and pathological management of body weight, the occurrence of which usually commences during adolescence and the early stages of adulthood (124, 137).

Anatomical, psychological, societal, and traditional influences are fundamental factors contributing to the increased incidence of EDs (138). About 5% of the general population suffers from EDs (139). However, several studies stated that athletes are more vulnerable to EDs than non-athletes, due to some sports-specific attributes and constraints from the coaches, teammates, and sports community that accentuate an idealized body shape, leanness, or low body weight aiming to improve athletic performance (124, 137, 140). Besides, revealing sports attire, esthetic evaluations, and weight restrictions are distinctive factors experienced by athletes and have been linked to DE behaviors. Anorexia athletica and bigorexia are kinds of DE habits related to athletes (141, 142).

Interestingly, a cutting-edge global meta-analysis review by Ghazzawi et al. (143), involving over 70,000 athletes of all ages, sport types, and levels of competition, reported that 19% (1 out of 5) athletes were engaged in DE behaviors such as self-induced vomiting, purging, excessive exercising, and dietary restrictions, especially in endurance, esthetic, and weight-dependent sports.

Also, the prevalence of ED among 611 first-year adolescent elite athletes in different disciplines and 355 non-athletes who participated from various Norwegian elite sports high schools was meaningfully higher, 7.0% in athletes compared to 2.3% in controls, representing a difference of 4.7% (144). On the other hand, adolescents and young elite athletes may be more exposed to developing EDs since they are already at a critical developmental stage, in which they encounter both general and sports-related risk factors, as well as being in the well-known period of typical onset of EDs in the overall population (132, 145).

Unfortunately, we found limited studies that involved junior athletes’ EDs symptoms, where symptoms were often assessed using questioners such as the Eating Disorder Examination-Questionnaire (EDE-Q), the Eating Disorder Inventory-I/II (EDI) Questionnaire, and the Eating Attitudes Test (EAT-26). These types of self-reported techniques are in doubt regarding their effectiveness and validity in reporting EDs among athletes (146). Furthermore, a lack of precise terminology for DE and the focus of most of these studies on specific groups (males or females) from particular types of sports or not including non-athletic participants from the same age group being studied make it difficult to reach a definite and clear result about this subject (145, 147, 148).

Disturbed eating behaviors, along with weight and negative body perception, were generally approved as key features that considerably affect the occurrence of EDs among junior elite athletes, leading to unfavorable outcomes (149, 150). Elite athletes who compete at high levels are more subjected to strenuous training regimens to adhere to ideal body appeal and weight (150–152). Consequently, they may view certain eating behaviors, such as fast weight loss or muscle gain, perspiration, and dietary restriction, as integral parts of a dedication to their sports and to athletic triumph. Hence, the detection of DE behaviors is more challenging in this category, and underreporting of EDs usually occurs (148, 151).

Mostly, EDs rates were highest among female athletes compared to male athletes (143, 153). In a study by Teixidor-Batlle et al. (154) approximately 5% (7.6% of females and 2.5% of male athletes) of 646 Spanish elite athletes with a mean age of 16.7 demonstrated the possibility of having EDs, especially among male endurance athletes and esthetic female athletes. In agreement with this study, a total of 583 athletes and non-athlete adolescents aged from 15 to 17 were selected to investigate the potential for EDs. The at-risk group consisted of a greater ratio of girls than boys (1:3.5) and a higher number of individuals with higher BMI percentiles (152). Conversely, there was no association between gender and the prevalence of EDs and DE among junior athletes (138, 148).

Additionally, 11% of junior and senior beach handball elite female athletes aged from 15 to 35 years were at higher risk of developing an ED, compared to 3% of male athletes in the same age group. Also, there was a positive correlation between BMI and the EAT-26 total score in females, while male athletes were negatively associated with BMI (124).

The connections athletes have with social media, idols in the sport, coaches, teammates, friends, and family in conjunction with economic status, ethnicity, and perfectionism can engender the onset of risk factors associated with ED (149, 155).

Eating disorders can have a negative impact and irreversible consequences on an individual’s overall health, psychological well-being, educational and sports performance, self-admiration, concentration, and social connections. Furthermore, it may lead to menstrual irregularities (amenorrhea) and bone demineralization, higher rates of anxious-depressive symptoms, a history of psychological, physical, or sexual violence, social withdrawal, and self-doubt in elite athletes with EDs. Thus, there is an urgent need for validated tools specific to the early detection and diagnosis of EDs among junior elite athletes (137, 147, 148, 156).

## 4 Conclusion

Our narrative review study included 48 research articles involving 4,005 juniors and studied junior athletes’ nutritional needs, including the effects of nutrients and supplements on the performance of young athletes. Most studies showed that junior athletes do not adhere to healthy nutrition plans, are exposed to nutrient and energy deficiency, and are more prone to eating disorders. The study concludes that macronutrients are crucial for junior athletes and the requirements vary according to exercise level, age, and sex. Physiological growth, energy metabolism, immune function and performance may be affected by suboptimal nutritional status among athletes.

The CHO is a fundamental component of providing young athletes with energy for growth and development. Moreover, it is crucial to improve cognitive function and the main source of energy for junior athletes’ muscles and the favored fuel for the brain and nervous system. Approximately 600 g of carbohydrate pre-match should be given 36 h even the acute carbohydrates (7% CHO) in amounts of 250 mL 30 and 60 min before the game has improved the muscle with glycogen, which is essential to high performance, stimulates energy production, reduces muscle fatigue, and increases the ability to maintain concentration during the exercise. Additionally, consuming high-glycemic index foods within 30 min to 2 h immediately after exercise accelerates recovery and restores depleted glycogen stores. Especially, foods containing simple sugars such as fructose (fruits) and galactose (milk and dairy products) replenish glycogen stores faster than glucose content. Carbohydrate supplementation may be beneficial in prolonged matches with multiple bouts within a 24-h timeframe.

Protein is needed to build junior muscle, repair, recover, grow, develop, overall athletic performance, enhance exercise training, meet increased energy demands, also to get iron intake. Furthermore, combining CHO and proteins immediately after training reduced the body’s muscle protein breakdown.

Fat contributes to normal body function and healthy growth maturation in young athletes’ dietary fat is considered a source and absorption factor of fat-soluble vitamins (A, K, D, E), which is essential for facilitating hormone synthesis, so the deficiency of fat intake reduces exercise performance. The pre-workout meal should be low in fat to minimize gastric upsets, which reduce performance.

Depending on the type of exercise, in young athletes, some nutrients may be susceptible to deficiency such as CHO, also magnesium, zinc, iron, calcium, vitamin E, and vitamin D. Our study concluded that supplements were popular among juniors and the protein-based supplements and multivitamins, were the most prevalent among male whereas caffeine anhydrous among females. Young athletes should take care of calcium and vitamin D, particularly among individuals who lack sufficient exposure to sunlight to maintain bone mineral density because adequate intake is essential for reducing the risk of deficiency, muscle cramps and fracture risk in young athletes. Also juniors should take an adequate intake of iron to avoid common anemia, especially among adolescent girls.

A negative body image perception, and pathological management of body weight (i.e., fast weight loss, perspiration, and dietary restriction) as integral parts of a dedication to sports and athletic triumph, the occurrence of which usually commences during adolescence and the early stages of adulthood can lead to eating disorders and disturbed eating patterns which are popular among junior athletes, especially adolescent girls which can have negative side effects and irreversible consequences on an individual’s overall health, psychological well-being.

The clinical trials of nutrients, supplements, and eating disorders on junior athletes are limited. In the future, we recommend studying wide consideration of hydration status, supplements, wholesome food, food choices, and eating disorders for juniors. Also, improvements in validated tools specific to the early detection and diagnosis of eating disorders among junior elite athletes.
